# Is an Inquisition Warranted in Light of the Widespread Usage of Antibiotics in Dentistry?

**DOI:** 10.7759/cureus.70961

**Published:** 2024-10-06

**Authors:** Pankaj Dhawan, Janvi Kalra

**Affiliations:** 1 Department of Prosthodontics and Implantology, Manav Rachna Dental College, Faridabad, IND; 2 Department of Prosthodontics, Manav Rachna Dental College, Faridabad, IND

**Keywords:** antibiotics awareness, antibiotics therapy, antimicrobial resistance (amr), hospital dentistry, overuse of antibiotics

## Abstract

The overuse of antibiotics has caused antibiotic-resistant bacteria to develop, making it increasingly difficult to treat infections. The consequences of antibiotic resistance are significant and far-reaching. Antibiotic resistance leads to longer hospital stays, higher healthcare costs, and a decreased ability to undergo surgeries and other medical procedures that rely on antibiotics to prevent infections. This threatens the development of new antibiotics, as pharmaceutical companies are less likely to invest in drugs that may be less effective due to resistance.

We need to work together to prevent antibiotic resistance. Healthcare professionals and the general public need to be aware of the dangers of overusing and misusing antibiotics, and we need to impress upon the government that incentives and support be provided to the industry to help develop new antibiotics. We need to take action now to prevent the spread of drug-resistant bacteria and protect public health.

## Editorial

The transformative power of antibiotics in healthcare cannot be overstated. By preventing life-threatening infections and improving patient outcomes, they have become a cornerstone of modern medicine [1]. However, the improper use of antibiotics poses a significant and growing threat to both individual and public health. Misuse such as overuse, inconsistent administration, self-medication, and sharing of antibiotics has contributed to an alarming increase in resistance to antibiotics [2]. While dentistry’s role in this issue has been underexplored, evidence from several studies indicates an increase in antibiotic prescriptions by dentists, often involving broad-spectrum antibiotics without clear indications of bacterial infection. This concerning trend accelerates the development of antibiotic resistance, posing a critical challenge for healthcare systems globally.

Despite global variations in guidelines, most dental infections can be effectively treated with local interventions, and antibiotics should be reserved for only spreading infections. In the UK, over 70% of antibiotics were prescribed without concurrent dental treatment, with 81% not adhering to national guidelines. Similar trends have been observed in the United States and Australia, where a significant proportion of antibiotic prescriptions were deemed unnecessary or outside established guidelines [3].

In India, the world’s largest consumer of antibiotics, there has been widespread documentation of poor prescribing practices, particularly the inappropriate use of broad-spectrum antibiotics without bacterial confirmation. A combination of factors, including patient expectations, refusal of dental treatments, dentists’ preferences, and time constraints, contributes to the overprescription of antibiotics in primary dental care. This evidence underscores the urgency for directed involvement to improve antibiotic stewardship in dentistry [4].

Antibiotic resistance arises when bacteria evolve mechanisms to withstand the effects of antibiotics, resulting in previously effective treatments being ineffective. This resistance develops through mutations in genetics or through the acquisition of resistance genes from other bacteria [5]. As a result, infections that were once easily treatable become more difficult, if not impossible, to manage. Patients infected with antibiotic-resistant organisms often experience more severe symptoms, prolonged recovery times, and an increased risk of mortality due to limited treatment options.

The misuse of antibiotics in dentistry exacerbates this issue. The emergence of "superbugs" in dental settings, where antibiotics are inappropriately prescribed, represents a serious public health risk. With antibiotic resistance continuing to rise, the need for proactive measures to curb misuse and promote appropriate antibiotic prescribing has never been more urgent.

In response to the growing concern of antibiotic resistance, global organizations, such as the World Health Organization (WHO), the Centers for Disease Control and Prevention (CDC), and the Indian Council of Medical Research (ICMR), have developed certain norms to promote the appropriate use of antimicrobial agents. Antimicrobial Stewardship (AMS) programs, initially focused on hospital settings, are now being extended to outpatient care, including dental practices. Dentists are responsible for approximately 10% of global antibiotic prescriptions, much of this occurring in outpatient settings. Retrospective studies indicate that as much as 80% of antibiotics prescribed in dental practices are inappropriate, often deviating from CDC guidelines [3].

AMS programs aim to optimize antibiotic use and preserve their effectiveness over the long term. These programs focus on ensuring that antibiotic therapy is appropriate to the patient’s clinical condition and then verify that the prescribed drug, dose, and duration are correct. The CDC has developed the Core Elements of Outpatient Antibiotic Stewardship, which include committing to improving antibiotic prescribing practices, implementing evidence-based policies, monitoring prescribing behaviors, providing clinician feedback, and offering education and expertise.
